# Duodenal pyloric gland adenoma with a long pedicle causes intestinal obstruction: A case report

**DOI:** 10.1097/MD.0000000000043331

**Published:** 2025-07-11

**Authors:** Lvcong Zhu, Saihua Lv, Yaqun Li, Kelin Yao

**Affiliations:** a Department of Radiology, The Affiliated Hospital of Shaoxing University (Shaoxing Municipal Hospital), Shaoxing, Zhejiang Province, China; b Department of Gastrology, The Affiliated Hospital of Shaoxing University (Shaoxing Municipal Hospital), Shaoxing, Zhejiang Province, China; c Department of Pathology, The Affiliated Hospital of Shaoxing University (Shaoxing Municipal Hospital), Shaoxing, Zhejiang Province, China.

**Keywords:** case report, computed tomography, endoscopic, pathological symptoms, pyloric gland adenoma

## Abstract

**Rationale:**

The diagnosis of pyloric gland adenoma (PGA) can be challenging when a small lesion grows in an uncommon site, which has no characteristic imaging manifestations. However, radiological examination has specific value in the diagnosis of complications. PGA has a clear malignant potential, and its treatment depends on the size, shape, and location of the lesion. Minimally invasive endoscopic resection is an effective treatment option for this condition. This report aims to raise awareness among clinicians regarding such clinical scenarios.

**Patient concerns:**

In the current report, we present the case of a 28-year-old male patient with a duodenal pyloric adenoma with a long pedicle of >8 cm, causing intestinal obstruction and intermittent gastrointestinal bleeding. Finally, the tumor was completely removed endoscopically.

**Diagnoses:**

The pathological diagnosis result was (duodenum) pyloric gland adenoma, with some glands exhibiting mild to moderate dysplasia.

**Interventions:**

The patient underwent a hot trap resection of duodenal long-pedunculated polyps combined with nylon ropes and titanium clips. The operation was carried out under general anesthesia and lasted approximately 40 minutes, excluding the waiting time for anesthesia recovery. The tumor was discolored due to ischemia and was resected using high-frequency electrical resection.

**Outcomes:**

The patient was discharged on the 7th day after polyp resection. The patient recovered well after surgery and no recurrence was observed during follow-up.

**Lessons:**

PGAs are usually solitary in the gastric body and rarely occur in the duodenum, and the appearance of a long pedicle have rarely been reported. Considering that PGA has definite malignant potential and may cause intestinal obstruction, early diagnosis and minimally invasive endoscopic resection are conducive to good prognosis.

## 1. Introduction

Pyloric gland adenoma (PGA) is a rare subtype of gastric adenomas. PGAs account for 2% to 2.7% of gastric adenomas.^[1]^ Elster first reported PGA in 1976.^[2]^ In recent years, with the development of endoscopic technology, PGA have received increasing attention. PGA often occurs in older women and is associated with autoimmune gastritis (AIG), *Helicobacter pylori* (HP), and chemical gastritis.^[3]^ PGAs are usually solitary in the gastric body, but can also occur in other parts of the body and are mainly associated with pyloric gland metaplasia.^[4,5]^ PGA is a precancerous lesion that can evolve from low-grade to high-grade intraepithelial neoplasia and adenocarcinoma, with a reported cancer rate of 12% to 47%.^[6]^ Early detection and surgical resection are important in patients with PGA. However, PGA have no characteristic imaging features. Computed tomography (CT) is helpful for the diagnosis of PGA with intestinal obstruction or other intestinal tumors. However, the final diagnosis of PGA depends on pathological examination. Here, we describe a case of intestinal obstruction caused by duodenal PGA with a long pedicle in a young man.

## 2. Case report

**Figure 1.**
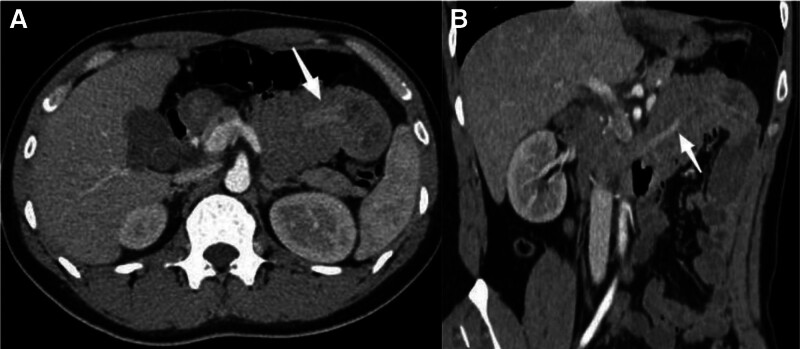
Arterial-phase CT image showing long strip-shaped abnormally enhanced shadows in the duodenum, accompanied by obstruction and dilation of the horizontal and jejunal segments of the duodenum. CT = computed tomography.

**Figure 2.**
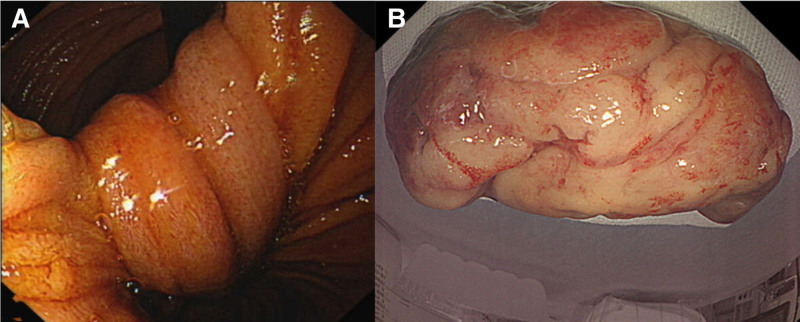
Endoscopic view of congestive long pedicle vegetation (A). The gross specimen was resected under the endoscope (B).

**Figure 3.**
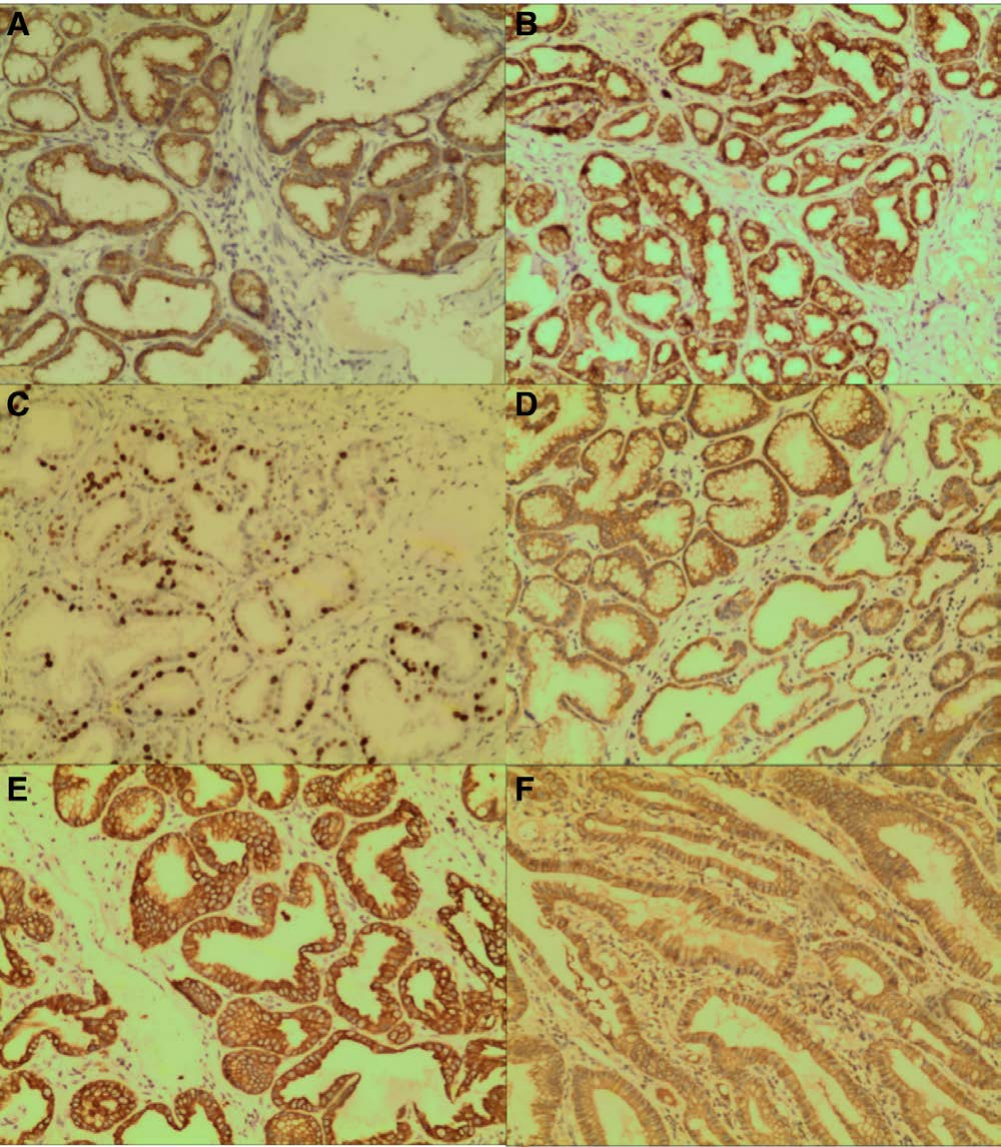
Immunohistochemical staining (100×): (A) MUC5AC(+), (B) MUC6(+), (C) Ki67(20%+), (D) EMA(+), (E) CK7(+), (F) Cad(+). MUC5AC = mucin apoprotein 5AC.

A 28-year-old male patient was admitted to the hospital following a 10-day history of upper abdominal pain and melena. The patient began to appear black and formed stool more than 10 days prior without obvious inducement. The frequency was once every 1 to 2 days. The stool had no mucus, pus, or blood. The patient had epigastric pain after eating and the symptoms had recently worsened, and the patient had no history of AIG, familial adenomatous polyps, or *Helicobacter pylori* infection. The patient had no history of diabetes, tuberculosis, hypertension, or hepatitis. The patient had no history of surgery or allergic drug use. The patient’s parents and sisters were healthy. The patient had no family history of gastric or colon cancers. No abnormalities were observed during the physical examination, and the patient underwent thorough evaluations, including routine investigations of blood, urine, feces, occult blood, blood biochemistry, and common serum tumor markers, such as carcinoembryonic antigen, carbohydrate antigen 19-9, alpha-fetoprotein, and carbohydrate antigen 724. No significant abnormalities were observed in any of the patients. To investigate the etiology of gastrointestinal bleeding in this young individual, an abdominal contrast-enhanced CT scan was performed in accordance with the clinical guidelines for non-variceal upper gastrointestinal bleeding. Complete abdominal enhanced CT revealed long strip-shaped abnormally enhanced shadows in the duodenum, accompanied by obstruction and dilation of the horizontal and jejunal segments of the duodenum (Fig. 1). Gastroscopy revealed chronic superficial gastritis without *Helicobacter pylori* infection in the background mucosa. Gastroscopy revealed a long-pedicle polypoid bulge in the duodenum (Fig. 2A). The pedicle length was >8 cm. The final diagnosis was (duodenum) pyloric gland adenoma, with some glands exhibiting mild-to-moderate dysplasia. Immunohistochemical staining (Fig. 3) revealed CEA(partial+), EMA(+), Cad(+), CK20(small amount+), CK7(+), P53(small amount of weak+), CDX2(partial+), Ki67(20%+), C-erbB2(1+), MUC2(small amount+), mucin apoprotein 5AC (MUC5AC)(+), and mucin apoprotein 6 (MUC6)(+).

The patient underwent a hot trap resection of duodenal long-pedunculated polyps combined with nylon ropes and titanium clips. The operation was carried out under general anesthesia and lasted approximately 40 minutes, excluding the waiting time for anesthesia recovery. During the surgery, the surgeon found a long peduncle vegetation extending from the posterior wall of the duodenal bulb to the descending part of the duodenum. The head was approximately 5 × 2 cm in size, with a nodular and cauliflower-like shape, and the surface was hyperemic and erosive (Fig. 2B). The head was embedded in the descending part of the duodenum and the endoscope could not pass through. Two nylon ropes were used to tighten the root and 3 titanium clips were used to reinforce the tumor. The tumor was discolored due to ischemia and was resected using high-frequency electrical resection. The tumor was removed from the mouth through the stomach and esophagus.

The patient recovered well after surgery, and no recurrence was observed during the follow-up.

## 3. Discussion

PGA is an uncommon gastrointestinal lesion with a significant malignant potential. It was included in the World Health Organization classification of gastric tumors in 1990. PGA often occurs in older women. However, in this case, the patient was a young man. PGA often occurs in the form of gastric polyps and can also occur in the duodenum, esophagus (Barrett esophagus), bile duct, gallbladder, pancreas, small intestine, rectum, and cervix and is mostly related to pyloric glandular metaplasia.^[7]^ In the present case, the lesion was located in the duodenum. PGA is often associated with AIG, HP, and chemical gastritis.^[8]^ This patient had no history of AIG or familial adenomatous polyps, and no positive history of HP. Previous studies have shown that MUC6 and MUC5AC have unique immunohistochemical expression patterns that are helpful in the diagnosis of PGA.^[9]^ Immunohistochemical analysis of the lesion in this patient revealed that the specimen was immunopositive for MUC6 (a pyloric gland mucin marker) and MUC5AC (a foveolar mucin marker), consistent with previous studies. In addition, positive expression of CDX2 and MUC2 in tumor cells was observed only in some areas, indicating that PGA mainly expressed gastric markers, intestinal epithelial differentiation was rare, and only a small amount of gastric-to-intestinal differentiation transition was observed. In this patient, CK7 and CK20 exhibited partial expression in certain regions. The specimen was also positive for p53 (a small amount of weak staining), and the Ki-67 level was >20%. It has been reported that the expression of p53 gradually increases in PGA from high-grade intraepithelial neoplasia to adenocarcinoma, and high p53 expression may indicate the relatively high carcinogenic potential of PGA. Additionally, pyloric adenomas tend to have a higher Ki-67 index when they have an adenocarcinoma component.

PGA usually shows flat, polypoid, or mass lesions, but some show irregular lesions with small volumes, and the appearance of a long pedicle has rarely been reported.^[10]^ In this case, the lesion presented as a rare, long pedicle with a length of more than 8 cm, which has not been previously reported. We hypothesized that the long pedicle structure makes the adenoma very mobile. Therefore, the adenoma head is easily incarcerated during intestinal peristalsis and causes secondary gastrointestinal bleeding. In this case, PGA was observed growing on the posterior wall of the bulb and extending to the descending part of the duodenum, with its head embedded, under endoscopy. Two nylon ropes were first used to tighten the base of the PGA, causing it to gradually change color due to ischemia. Concerned that the nylon ropes might slip off during resection, 3 titanium clips were also used to fix the base. Then, a high-frequency electrosurgical knife (50 W, electrocautery mode) was used to cut off the base of the PGA. After confirming no significant bleeding from the wound, the resected polyp was slowly withdrawn through the mouth as the endoscope was withdrawn.

In conclusion, the diagnosis of PGA can be challenging when a small lesion or lesion growing in an uncommon site has no characteristic imaging manifestations. However, imaging examination has a specific value in the diagnosis of complications. PGA has a clear malignant potential, and its treatment depends on the size, shape, and location of the lesion. Minimally invasive endoscopic resection is an effective treatment option for this condition.

## Author contributions

**Data curation:** Saihua Lv, Yaqun Li.

**Project administration:** Kelin Yao.

**Writing – original draft:** Lvcong Zhu.

**Writing – review & editing:** Kelin Yao.
